# Ethnic Disparities in Endothelial Function and Its Cardiometabolic Correlates: The Pathobiology of Prediabetes in A Biracial Cohort Study

**DOI:** 10.3389/fendo.2018.00094

**Published:** 2018-03-13

**Authors:** Ibiye Owei, Nkiru Umekwe, Hanan Mohamed, Sotonte Ebenibo, Jim Wan, Sam Dagogo-Jack

**Affiliations:** ^1^Division of Endocrinology, Diabetes, and Metabolism, University of Tennessee Health Science Center, Memphis, TN, United States; ^2^Department of Preventive Medicine, University of Tennessee Health Science Center, Memphis, TN, United States

**Keywords:** prediabetes, endothelial dysfunction, ethnic disparities, cardiometabolic risk, obesity, dyslipidemia

## Abstract

**Background:**

Endothelial function (EF) reflects the balance between vasodilatory and vasoconstrictive factors produced by (or acting on) the innermost lining of blood vessels. Endothelial dysfunction, an imbalance between these factors that favors vasoconstriction, has been associated with increased risk for cardiovascular disease. However, the influence of race/ethnicity and glycemic status on association between EF and cardiovascular risk factors remain to be clarified.

**Subjects and methods:**

We assessed EF in relation to glycemia and cardiometabolic profile in African-American (AA) and European-American (EA) offspring of parents with type 2 diabetes (T2D), who are participants in the prospective pathobiology and reversibility of prediabetes in a biracial cohort (PROP-ABC) study. Assessments at enrollment included a 75 g oral glucose tolerance test (OGTT), blood pressure, anthropometry, body composition (DEXA), and lipid profile. Other assessments were insulin sensitivity and resting energy expenditure. EF was measured using flow-mediated vasodilation (EndoPAT 2000) and expressed as reactive hyperemia index (RHI).

**Results:**

We studied 190 subjects (100 AA, 90 C), mean age (±SD) 53.1 ± 9.1 years, and body mass index 30.6 ± 6.8 kg/m^2^. Based on OGTT data, 96 subjects (52 AA, 44 EA) had prediabetes and 94 subjects were normoglycemic (48 AA and 46 EA). The RHI was lower in AA than EA (2.17 ± 0.55 vs. 2.36 ± 0.72, *P* = 0.05) and in prediabetic than normoglycemic subjects (2.14 ± 0.62 vs. 2.38 ± 0.65, *P* = 0.013). Using RHI ≤ 1.68 as diagnostic cut-off, 19% of participants with prediabetes and 10% of normoglycemic participants had endothelial dysfunction (*P* = 0.04). In univariate models, RHI was positively associated with age and HDL cholesterol levels, and inversely associated with adiposity, diastolic blood pressure, and 2hr plasma glucose. The association between RHI and adiposity was stronger in men than women. The association between RHI and age, glucose and HDL cholesterol displayed marked ethnic disparities.

**Conclusion:**

In our biracial cohort comprising offspring of parents with T2D, prediabetes increased the risk of endothelial dysfunction. However, the association between EF and cardiometabolic risk factors was significantly modified by ethnicity and gender. Our findings support current understanding of endothelial dysfunction as an early sensitive indicator of cardiometabolic risk.

## Introduction

Endothelium, the largest end organ in the body by surface area, is the target of numerous noxious stimuli, including proinflammatory cytokines, oxidative stress, shear stress from blood pressure, glycemia, and lipidemia, among others (1). Endothelial function (EF) reflects the balance between vasodilatory and vasoconstrictive factors produced by (or acting on) the innermost lining of blood vessels, and is traditionally assessed by methodologies based on measurement of flow-mediated vasodilation (2). Endothelial dysfunction, thus, is a pathological state that shifts the balance among the various vasoactive factors toward increased vasoconstrictive tone (2). Several reports have demonstrated that endothelial dysfunction is an early marker of cardiovascular disease in patients with diabetes (1–6). Indeed, endothelial dysfunction precedes fatty streaking, the well-known initiator of atherosclerosis and macrovascular disease (2, 4). Endothelial dysfunction also is increasingly being recognized as a complication of prediabetes (7–10).

However, the association between endothelial dysfunction and prediabetes has not been as well documented as that between endothelial dysfunction and established diabetes. Moreover, the influence of race/ethnicity on any association between endothelial dysfunction and prediabetes merits investigation. Indeed, the few studies on EF in prediabetic individuals are limited by lack of demographic diversity among participants (7, 8). The reported higher prevalence of diagnosed type 2 diabetes (T2D) and associated complications in African-Americans (AA) compared to European-Americans (EA) provide a rationale for investigating ethnic patterns in endothelial dysfunction in prediabetes. Furthermore, hyperglycemia is known to adversely affect EF (5, 6), which makes glycemic control a confounding variable when comparing EF across individuals with diabetes.

The pathobiology and reversibility of prediabetes in a biracial cohort (PROP-ABC) study is a prospective study of non-diabetic AA and EA offspring of parents with T2D. The cohort comprises participants with normal glucose values and those with recently recognized prediabetes. Data from our biracial PROP-ABC participants has enabled us to investigate the influence of ethnicity on EF before the development of diabetes and to document evidence of endothelial dysfunction in prediabetes. Studies have shown that lifestyle intervention (and certain medications) could prevent or delay the development of T2D among people with prediabetes (11–14). Thus, our study provides a framework for longitudinal assessment of the impact of interventions on the reversibility of prediabetes and early endothelial dysfunction.

## Materials and Methods

### Study Subjects

We studied AA and EA male and female offspring of parents with T2D, who are participants in the prospective PROP-ABC study. The latter is a continuation of the pathobiology of prediabetes in a biracial cohort (POP-ABC) (15, 16).

**Figure 1:**
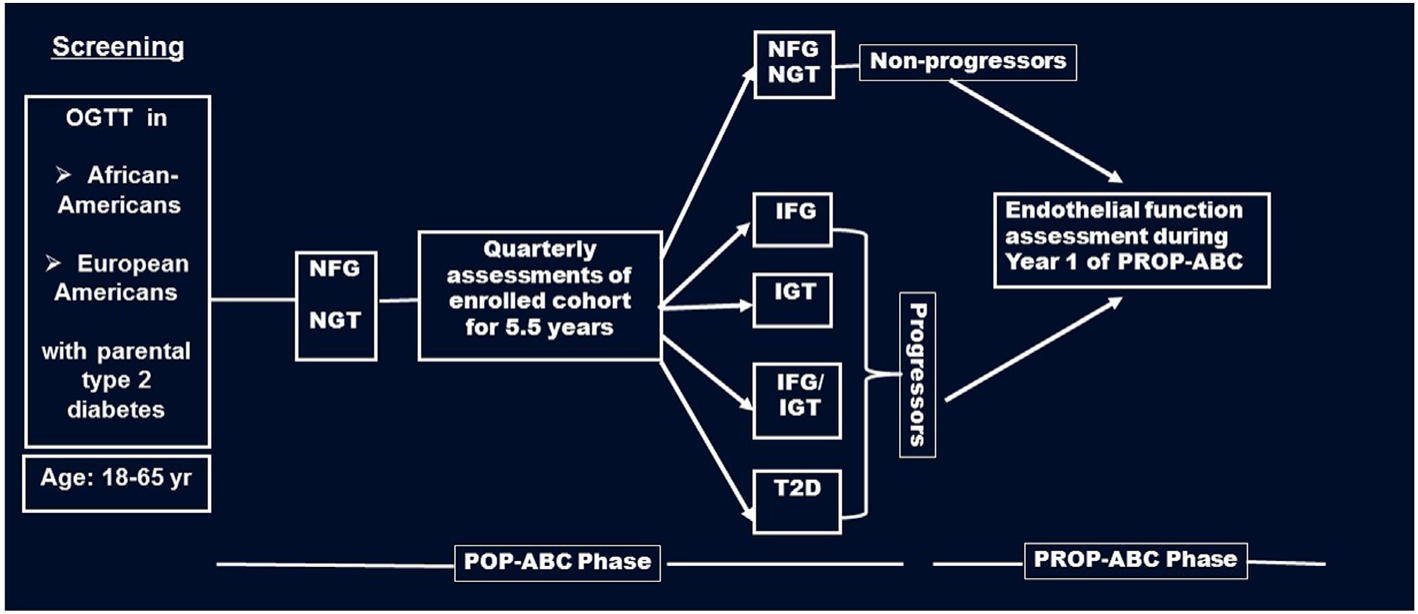
Flow diagram of screening, enrollment, and follow-up of participants in the pathobiology of prediabetes in a biracial cohort (POP-ABC) study leading to the transition to the pathobiology and reversibility of prediabetes in a biracial cohort (PROP-ABC) phase. Endothelial function was measured during year 1 of PROP-ABC as part of the baseline assessment of participants who completed the parent POP-ABC study (duration 5.5 years, mean follow-up 2.62 years) and agreed to re-enroll for the continuation PROP-ABC study.

Briefly, the POP-ABC study was a longitudinal study in which normoglycemic AA and EA offspring of individuals with T2DM were followed for the occurrence of incident prediabetes, defined as impaired fasting glucose (IFG) and/or impaired glucose tolerance (IGT) using American Diabetes Association criteria (17). The pre-specified age range for inclusion was 18–65 years. Subjects with a history of diabetes, major illnesses, recent hospitalization, and those using medications known to alter body weight or glucose metabolism were excluded from the POP-ABC study (15, 16). During 5.5 years of follow-up, 101 of 343 participants developed incident prediabetes and were notified without any interventions. The aim of the PROP-ABC study includes providing intensive lifestyle intervention to subjects with incident prediabetes, while continuing to follow those who remain normoglycemic. The present report compares EF and other parameters in 96 of the participants with incident prediabetes (Progressors) and a group of 94 participants matched with age, gender, and ethnicity who remained normoglycemic (Non-progressors). EF was measured during year 1 of PROP-ABC as part of the baseline assessment in participants who completed the parent POP-ABC study (duration 5.5 years, mean follow-up 2.62 years) and agreed to re-enroll for the continuation PROP-ABC study (Figure 1). Thus, the present report is a cross-sectional comparison of Progressors vs. Non-progressors at the end of POP-ABC study. The study protocol was approved by the University of Tennessee Institutional Review Board and written informed consent was obtained from all participants before initiation of the study, which was conducted in accordance with ethical principles established by the Declaration of Helsinki.

### Procedures and Measurements

The procedures at enrollment included a 75-g oral glucose tolerance test (OGTT), anthropometric and blood pressure measurements, lipid profile, and blood biochemistries. Blood pressure was recorded in the seated position, using an automated sphygmomanometer (Welch Allyn monitor, Skaneateles Falls, NY, USA); the average of two readings was used for calculations. The ankle-to-brachial blood pressure index (ABI) was calculated as the ratio of the mean systolic blood pressure measured over the posterior tibial artery in both lower extremities to the systolic blood pressure measured in the brachial vessel of the right arm. The average of two measurements taken from each location, 10 min apart, was used for calculating the ratio. Body weight (in light outdoor clothing) was measured in duplicate on a calibrated balance beam scale (Tanita scale, Arlington height, IL, USA). Standing height (without shoes) was determined in duplicate with a standard stadiometer. The body mass index (BMI) was calculated as weight in kilogram divided by the square of the height in meters. Waist circumference was determined to the nearest 0.1 cm at the midpoint between the highest point of the iliac crest and the lowest costal margin in the mid-axillary line, using a Gulick II tape measure. Total and trunk fat mass was measured using DEXA. Insulin sensitivity (Si-clamp) was measured using the hyperinsulinemic euglycemic clamp, as previously described (18). In brief, steady-state glucose infusion rate was expressed in molar units per kilogram of fat-free mass to derive metabolic clearance of glucose (M). The latter was then corrected for steady-state plasma insulin levels (M/I) to obtain insulin sensitivity values. Resting energy expenditure (REE) was measured using indirect calorimetry, as previously described (18).

#### Endothelial Function

Endothelial function was assessed using the well-validated EndoPAT 2000 device (ITAMAR Medical, Caesarea, Israel) and expressed as reactive hyperemia index (RHI) (19, 20). Participants underwent the procedure following a 12-h overnight fast, and were instructed to maintain usual diet, avoid tobacco or alcohol consumption, or unusual exercise prior to the test day. Briefly, overnight fasted subjects remained supine for 20 min in a quiet room before the test. A single-use sleeve is placed on the index finger of each hand to continuously measure peripheral arterial tone. The brachial artery in the upper arm of the non-dominant arm (test arm) was occluded with a blood pressure cuff for 5 min, followed by rapid release. The dominant arm without any manipulation served as the control. The EndoPAT 2000 software integrates inputs from the finger sleeves of the control and the test arms (at baseline, during occlusion, and after release) and generates data for flow-mediated vasodilatation (measured as RHI) (19–21).

### Statistical Analyses

All statistical analyses were conducted using SAS statistical software, version 9.3 (SAS Institute Inc., Cary, NC, USA). Data were reported as means ± SD unless otherwise specified. Differences between groups were analyzed using unpaired *t*-tests for discrete variables and χ^2^ test for ordinal variables. General linear regression models were used to compare anthropometric and cardiometabolic data in AAs and EAs and between prediabetic and normoglycemic subjects. The relationship between EF (RHI) and cardiometabolic markers (weight, BMI, waist circumference, total fat mass, trunk fat mass, cholesterol, and insulin sensitivity) was analyzed using linear regression and Pearson correlation coefficients. The level of significance was set at *P* < 0.05.

## Results

### Cohort Description

We studied 190 subjects (100 AA, 90 EA) who had complete results for EF. Of the 190 subjects, 96 (52 AA and 44 EA) were participants who developed incident prediabetes during follow-up (Progressors) and 94 (48 AA and 46 EA) were age-, gender-, and ethnicity-matched participants who maintained normoglycemia (Non-progressors). The mean age (±SD) was 53.1 ± 8.64 years in Progressors and 52.5 ± 9.56 years in Non-progressors (*P* = 0.36); the mean BMI 31.6 ± 7.00 kg/m^2^ in Progressors and 29.1 ± 6.33 kg/m^2^ in Non-progressors (*P* = 0.003).

The BMI was higher (32.5 ± 6.9 vs.28.5 ± 6.1 kg, *P* < 0.0001) and insulin sensitivity lower (0.12 ± 0.07 vs. 0.15 ± 0.07, *P* = 0.02) in AA than EA participants, but there was no difference in fasting plasma glucose (FPG), 2-hour plasma glucose (2hrPG), or REE between the two groups (Table 1). Total body fat mass, trunk fat mass, waist circumference, and blood pressure values were higher, but ABI and triglycerides were lower in AA compared to EA (Table 1).

**Table 1:** Baseline characteristics of study subjects.

	African-American (AA)	European-American (EA)	*P*-value
Number	100	90	
**Glycemic status**			
Prediabetes	52	44	
Normoglycemia	48	46	
Age (years)	51 ± 9.3	55 ± 8.5	0.003
BMI (kg/m^2^)	32.5 ± 6.9	28.5 ± 6.1	<0.0001
Waist (cm)	98.9 ± 15.9	91.7 ± 16.1	0.003
Total fat mass (kg)	38.3 ± 17.9	32.5 ± 12.7	0.02
Trunk fat mass (kg)	20.2 ± 8.8	16.9 ± 7.5	0.01
SBP (mmHg)	124 ± 14.1	119 ± 11.1	0.008
DBP (mmHg)	77 ± 8.9	75 ± 7.7	0.03
ABI	1.00 ± 0.1	1.10 ± 0.09	0.0002
FPG (mg/dl)	90.7 ± 8.2	92.1 ± 6.8	0.21
2 hrPG, mg/dl	139 ± 31.3	133 ± 30.2	0.18
LDL (mg/dl)	108 ± 30	107 ± 29.2	0.92
HDL (mg/dl)	53.8 ± 14.3	53.5 ± 13.1	0.89
Triglycerides (mg/dl)	81 ± 38.6	104 ± 45	0.0003
REE (kcal/kg FFM)	14.5 ± 0.4	14.2 ± 0.3	0.63
**Si-clamp**			
(μmol/kg FFM·min^−1^/pmol/L)	0.12 ± 0.07	0.15 ± 0.07	0.02

*ABI, ankle-to-brachial index; DBP, diastolic BP; FFM, fat-free mass; FPG, fasting plasma glucose; REE, resting energy expenditure; SBP, systolic BP; Si-clamp, insulin sensitivity measured by hyperinsulinemic euglycemic clamp; 2hrPG, two hour post-load plasma glucose during 75 g standard oral glucose tolerance test*.

### Endothelial Function

Endothelial function assessed by flow-mediated vasodilation (RHI) was similar in men and women, and tended to be lower in AA than EA subjects, although the difference was of marginal statistical significance (2.17 ± 0.55 vs. 2.36 ± 0.72, *P* = 0.05). In contrast, progressors to prediabetes had significantly lower RHI than Non-progressors who maintained normoglycemic during longitudinal follow-up (RHI: 2.14 ± 0.62 vs. 2.38 ± 0.65, *P* = 0.013) (Table 2). In a previous report, endothelial dysfunction was defined as the mean (2.34 ± 0.33) minus 2 standard deviations of RHI values in 20 healthy asymptomatic individuals without a history of cardiovascular disease or major risk factors, corresponding to RHI ≤ 1.68 (22). Using that cut-off RHI value of 1.68, we found that 19% of our participants with incident prediabetes (Progressors) had endothelial dysfunction as compared to 10% among Non-progressors (Chi squared *P* = 0.04).

**Table 2:** Endothelial function [reactive hyperemia index (RHI)] in relation to glycemic status, gender, and ethnicity among offspring of parents with type 2 diabetes.

Category	RHI	*P*-value
Progressors (prediabetes)	2.14 ± 0.62	
Non-progressors (normoglycemia)	2.38 ± 0.65	0.01
Male	2.19 ± 0.64	
Female	2.17 ± 0.55	0.37
African-American	2.29 ± 0.65	
European-American	2.36 ± 0.72	0.05

### Correlates of EF

**Figure 2:**
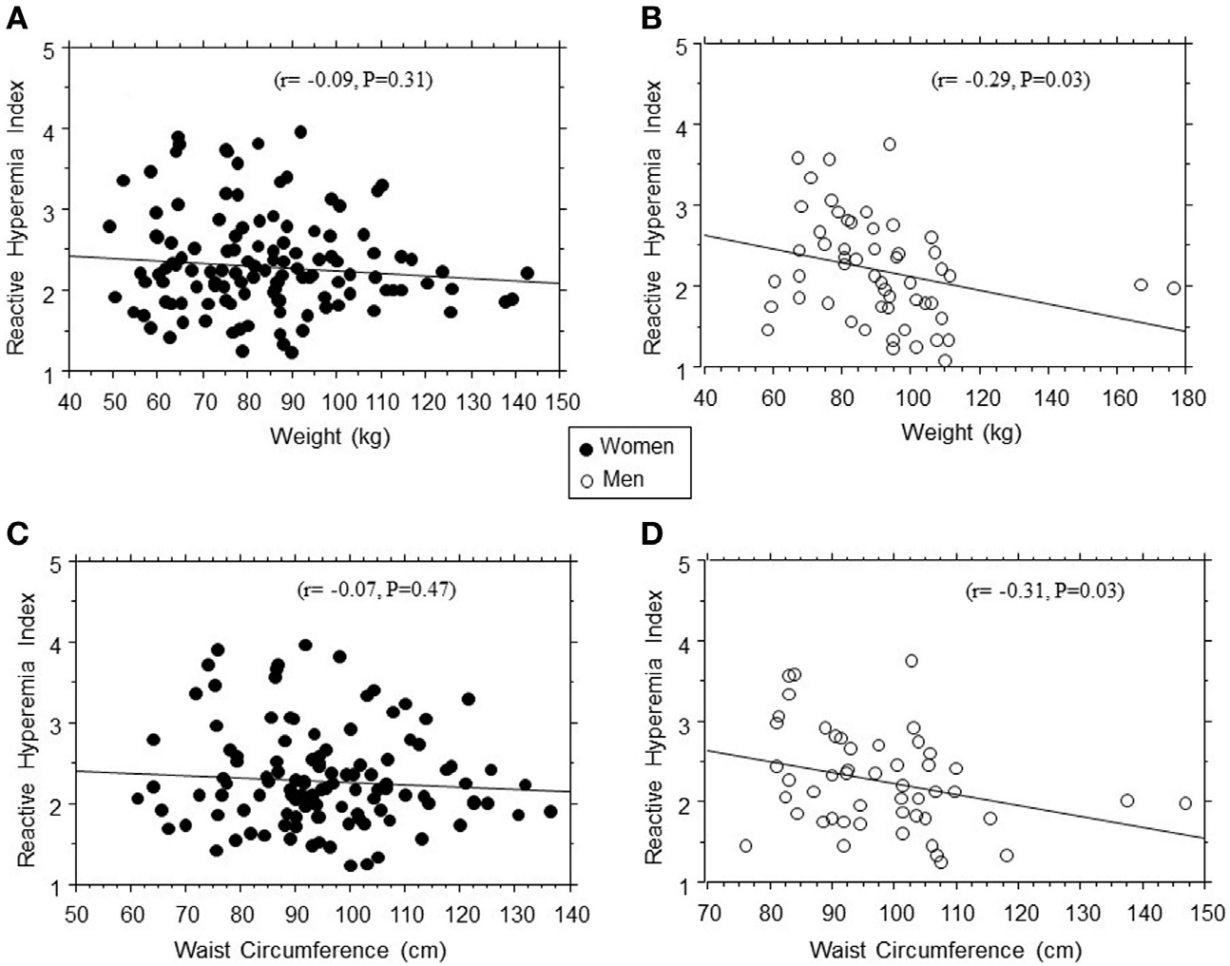
Regression plots of endothelial function (reactive hyperemia index) vs. weight **(A,B)** and waist circumference **(C,D)** in women (closed circles) and men (open circles).

In linear regression models, RHI was positively associated with age (*r* = 0.21, *P* = 0.005) and inversely associated with weight (*r* = −0.16, *P* = 0.03), BMI (*r* = −0.12, *P* = 0.08), diastolic blood pressure (*r* = −0.14, *P* = 0.06), and 2hrPG (*r* = −0.22, *P* = 0.04) in the entire study population. The association between RHI and body weight was stronger in men (*r* = −0.29, *P* = 0.003) than women (*r* = 0.08, *P* = 0.42) (Figure 2). Other adiposity measures displayed a similar gender pattern in their association with RHI: waist circumference (*r* = −0.31, *P* = 0.03 in men vs. *r* = −0.07, *P* = 0.46 in women); total fat mass (*r* = −0.23, *P* = 0.05 in men vs. *r* = −0.08, *P* = 0.37 in women); and trunk fat mass (*r* = −0.19, *P* = 0.06 in men vs. *r* = −0.07, *P* = 0.42 in women).

**Figure 3:**
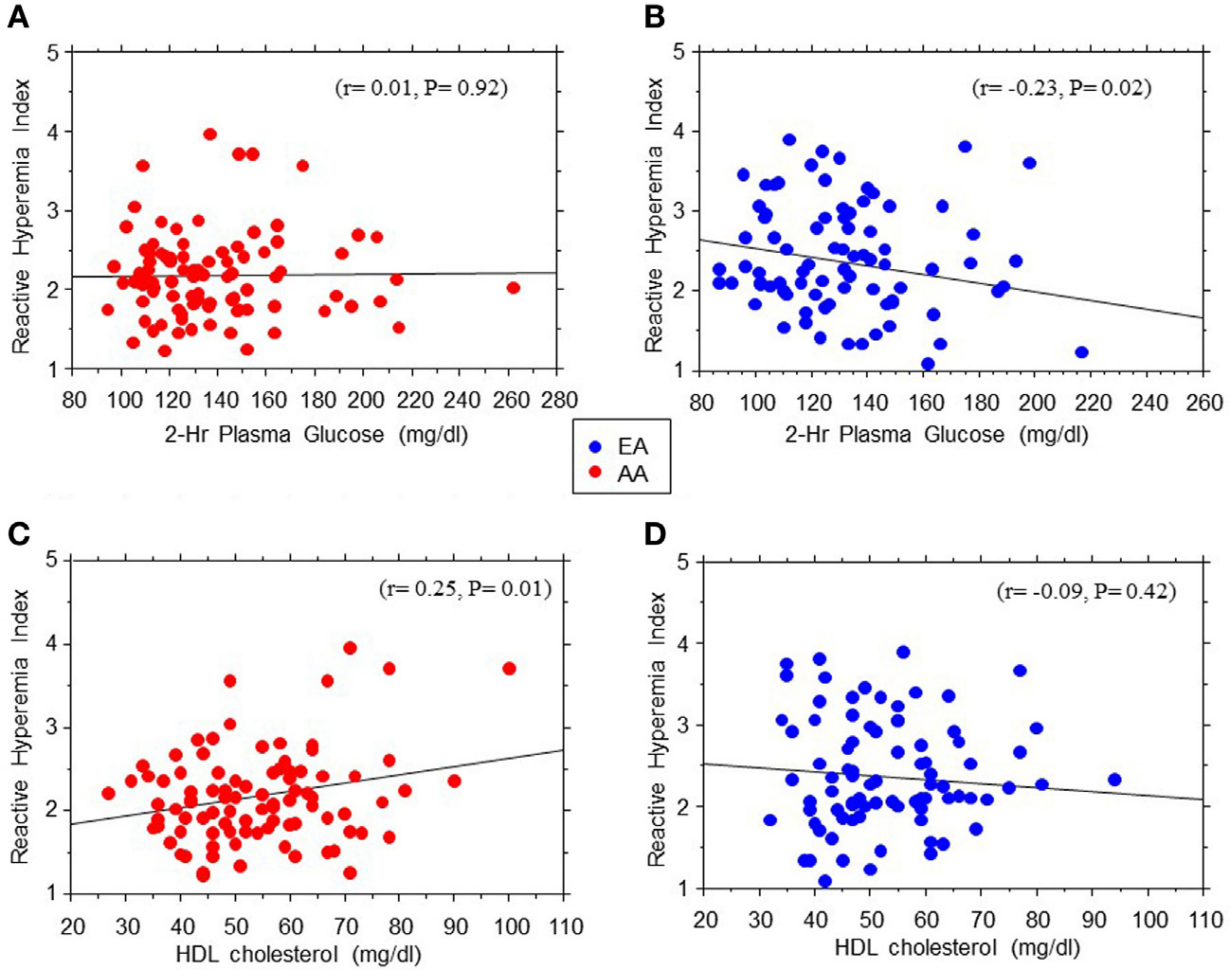
Regression plots of endothelial function measured as reactive hyperemia index vs. 2hr post-load plasma glucose levels during oral glucose tolerance test **(A,B)** and HDL cholesterol **(C,D)** in African-American (AA, red symbols) and European-American (EA, blue symbols) offspring of parents with type 2 diabetes.

**Figure 4:**
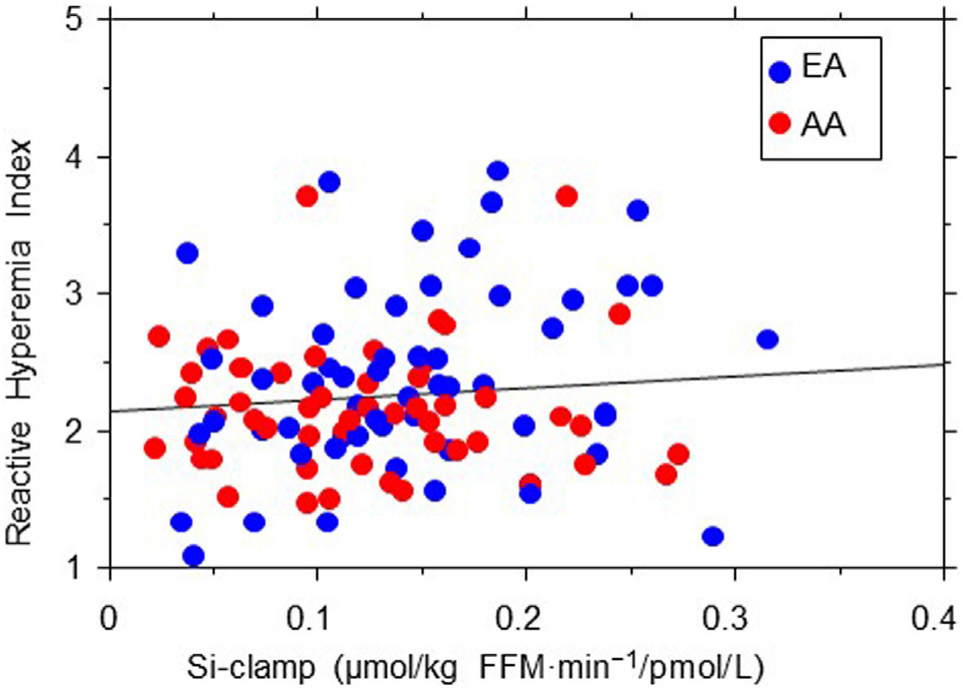
Regression plot of endothelial function (reactive hyperemia index) vs. insulin sensitivity (Si-clamp) in African-American (AA, red symbols) and European-American (EA, blue symbols) offspring of parents with type 2 diabetes. There was no significant correlation between reactive hyperemia index and Si-clamp in AA (*r* = 0.01, *P* = 0.97) or EA (*r* = 0.1, *P* = 0.40) subjects.

The association between EF (RHI) and age seemed stronger in AA than EA subjects (*r* = 0.26, *P* = 0.012 in AA vs. *r* = 0.13, *P* = 0.1 in EA). Similarly, we found significant Black–White differences in the strength of association between EF and 2hrPG (*r* = 0.01, *P* = 0.92 in AA vs. *r* = −0.23, *P* = 0.02 in EA) and HDL cholesterol levels (*r* = 0.25, *P* = 0.014 in AA vs. *r* = 0.09, *P* = 0.42 in EA) (Figure 3). No associations were observed between RHI and fasting plasma glucose, HbA1c, systolic blood pressure, total cholesterol, triglycerides, ABI, Si-clamp, or REE in our biracial cohort of healthy offspring of parents with T2D. Specifically, the correlation coefficient between RHI and Si-clamp was 0.01 (*P* = 0.97) in AA and 0.1 (*P* = 0.40) in EA (Figure 4). Similarly, the correlation coefficient between RHI and REE was 0.08 (*P* = 0.45) in AA and 0.09 (*P* = 0.43) in EA.

## Discussion

Endothelial dysfunction is an early marker of cardiovascular disease that precedes fatty streaking during the development of atherosclerosis (23, 24). Our findings of significantly lower EF in prediabetic individuals compared to normoglycemic control is consistent with the report by Gupta et al. on the relationship between dysglycemia and EF (7). Compared with lean normoglycemic subjects, age-matched obese individuals with prediabetes (FPG 106 mg/dl) showed evidence of endothelial dysfunction (as indicated by impaired flow-mediated dilatation) (7). Consistent with our data, an inverse relationship between EF and adiposity was also noted in prediabetic individuals (7). In a previous report, endothelial dysfunction was defined as the mean RHI (2.34) minus 2 SDs (0.33) obtained from 20 healthy asymptomatic individuals without a history of cardiovascular disease or major risk factors, yielding a cut-off values of ≤1.68 (23). Based on that criterion, 19% of our subjects with prediabetes, as compared to 10% of normoglycemic participants, had endothelial dysfunction. These findings support the notion that the prediabetes milieu is toxic environment for the initiation of vascular complications (9).

Measures of adiposity were inversely related to EF, as measured by RHI in this study. Obesity is associated with the activation of proinflammatory cytokines, lower adiponectin levels, insulin resistance, and dysglycemia, factors that bode ill for EF (1–8). In this study, we observed that the association between EF and adiposity measures was much stronger in men than women. Our findings are consistent with a recent report by Dass et al. that brachial artery flow-mediated dilation was significantly, inversely associated with total and central adiposity measures in men but not in women, among 108 healthy AA nonsmokers aged 18–45 years (22). As we found no difference in mean RHI between men and women in this study, the modifying effect of gender was expressed as a relative flattening of the effect of increasing adiposity on RHI in women (vs. declining RHI in men, Figure 1). Together, our findings and the related report by Dass et al. indicate a modifying effect of gender on the interaction between EF and adiposity. The plausible mechanisms likely involve a protective effect of estrogen that somehow blunts the deleterious effect of obesity on the endothelium (25–27), although a deleterious effect of androgen may also be argued.

The mean RHI was numerically lower in AA participants compared to their white counterparts, with a borderline statistical significance (*P* = 0.05). Other workers have reported significant ethnic disparities in EF, driven in part by greater adiposity and cardiometabolic risk factors in AAs (28). In addition to greater adiposity, other plausible mechanisms for the lower EF in AAs may relate to a higher burden of proinflammatory cytokines and lower adiponectin levels (29–32). Interestingly, we observed a positive association between RHI and age, especially among AA participants, which would seem paradoxical, as older age is known to be associated with greater cardiovascular disease burden. However, the association between age and EF (measured by flow-mediated dilation) is rather complex and subject to modification by lifestyle factors (7, 33, 34). In one report, BMI-adjusted RHI was not correlated with age (7). We also observed marked ethnic differences in the associations between EF (RHI) and 2-hrPG and HDL cholesterol levels. The significant association between RHI and 2-hrPG noted in EA participants was not evident among AA participants. In contrast, the robust association between RHI and HDL cholesterol levels observed in AA subjects was absent among EA participants.

The mechanisms for these disparate associations between EF and glucose or HDL cholesterol levels are unclear. Hyperglycemia impairs EF, in part, by activating reactive oxygen species that can negate the beneficial vasodilatory effects of endothelium-derived nitric oxide (35). In contrast, HDL and its associated paraoxonase exert anti-inflammatory and anti-oxidant properties that are vasoprotective (36, 37). Thus, the observed ethnic differences in the associations between EF and glycemia or HDL cholesterol might indicate differences in HDL functionality or sensitivity to glucotoxicity (38, 39). In terms of integrated physiology, flow-mediated vasodilation (expressed as RHI) is a surrogate measure of endothelial nitric oxide synthase (eNOS) activation and endothelial nitric oxide release, leading to vasodilation (40). Although eNOS plays a central role in the control of endothelial homeostasis, acetylcholine released by endothelial cells is an important component of the flow-mediated vascular response (41). Once released, acetylcholine mobilizes calcium from intracellular compartment within endothelial cells and stimulates nitric oxide production, leading to vascular relaxation (41). Recently, telomerase activity has been shown to also modulate microvascular flow-mediated dilation through an ability to switch vascular signal from nitric oxide to toxic-free radicals, such as mitochondrial hydrogen peroxide in the setting of atherosclerotic cardiovascular disease (42).

We observed no significant correlation between EF (RHI) and insulin sensitivity in our EA or AA participants. As insulin-induced nitric oxide production from the endothelium increases blood flow and glucose uptake in skeletal muscle, a direct correlation between insulin sensitivity and EF can be expected (43). However, several studies have examined the relationship between insulin action and EF in humans, with variable results (44–48). In those studies, EF was assessed by measuring blood flow, and insulin action was assessed by using various methods (including hyperinsulinemic euglycemic clamp, minimal modeling, and homeostasis model of insulin resistance). Muniyappa and Sowers pooled the correlation coefficients between insulin sensitivity and EF for 12 such studies and reported a value of 0.14 (*P* = 0.001) (49). Thus, the correlation between insulin sensitivity and EF seems rather weak. Furthermore, components of the insulin resistance syndrome (including obesity, dyslipidemia, hypertension, dysglycemia, adipocytokine secretome, etc.) often are associated with alteration of EF. Consequently, multivariate analyses that controlled for potential confounders, usually report no significant relationship between insulin sensitivity and endothelial dysfunction (48–51). Also, the lack of a significant correlation between EF and insulin sensitivity in our study might be explained in part by the overall healthy blood pressure, lipid, and glycemic profile of the POP-ABC participants. It is plausible that a less healthy cohort could have generated a different pattern of relationship between EF and insulin sensitivity.

The strengths of our study include the large sample size, the largely operator-independent procedure for assessing EF, and utilization of a biracial cohort. Also, the study design of the parent POP-ABC study allowed us to study subjects with incident prediabetes detected during longitudinal follow-up. The limitations of our study include the lack of serial data on temporal changes in EF and our inability to account for behavioral and environmental factors that could affect EF. Despite these limitations, our findings are concordant with previous observations regarding correlates of endothelial dysfunction. Moreover, our study extends previous reports by demonstrating, in a biracial cohort comprising AA and EA offspring of parents with T2D, that prediabetes increased the risk of endothelial dysfunction. In conclusion, we have shown that endothelial dysfunction is a sensitive indicator of recent transition from normoglycemia to prediabetes status, thus reinforcing current understanding of the cardiovascular risk associated with prediabetes.

## Author Contributions

The authors’ individual contributions included the following: SD-J as the principal investigator, developed the study concept and design, and wrote the manuscript. IO collected the data, conducted the statistical analysis, drafted, reviewed, and revised the manuscript. NU collected the data, reviewed, and revised the manuscript. HM collected the data, reviewed, and revised the manuscript. SE reviewed and revised the manuscript. JW conducted statistical analysis, reviewed, and revised the manuscript.

## Conflict of Interest Statement

The author declares that the research was conducted in the absence of any commercial or financial relationships that could be construed as a potential conflict of interest.

## Abbreviations

ABI, ankle-to-brachial blood pressure index; AA, African-Americans; BMI, body mass index; DEXA, dual energy X-ray absorptiometry; EA, European-Americans; EF, endothelial function; FFM, fat-free mass; FPG, fasting plasma glucose; IFG, impaired fasting glucose; IGT, impaired glucose tolerance; OGTT, oral glucose tolerance test; POP-ABC, pathobiology of prediabetes in a biracial cohort; PROP-ABC, pathobiology and reversibility of prediabetes in a biracial cohort; REE, resting energy expenditure; RHI, reactive hyperemia index; Si-Clamp, insulin sensitivity measured by hyperinsulinemic euglycemic clamp; 2hrPG, two hour post-load plasma glucose during 75-g standard oral glucose tolerance test; T2D, type 2 diabetes.

## References

[1] Vita JA Keaney JF Endothelial function: a barometer for cardiovascular risk? Circulation (2002) 106:640–2. 10.1161/01.CIR.0000028581.07992.56 12163419

[2] Deanfield JE Halcox JP Rabelink TJ Endothelial function and dysfunction: testing and clinical relevance. Circulation (2007) 115:1285–95. 10.1161/CIRCULATIONAHA.106.652859 17353456

[3] Ylitalo KR Sowers M Heeringa S. Peripheral vascular disease and peripheral neuropathy in individuals with cardiometabolic clustering and obesity: National Health and Nutrition Examination Survey 2001–2004. Diabetes Care (2011) 34:1642–7. 10.2337/dc10-2150 21593304 PMC3120210

[4] Corrado E Rizzo M Coppola G Muratori I Carella M Novo S. Endothelial dysfunction and carotid lesions are strong predictors of clinical events in patients with early stages of atherosclerosis: a 24-month follow-up study. Coron Artery Dis (2008) 19:139–44. 10.1097/MCA.0b013e3282f3fbde 18418229

[5] Shachor-Meyouhas Y Pillar G Shehadeh N. Uncontrolled type 1 diabetes mellitus and endothelial dysfunction in adolescents. Isr Med Assoc J (2007) 9:637–40. 17939622

[6] Mahmud FH Earing MG Lee RA Lteif AN Driscoll DJ Lerman A. Altered endothelial function in asymptomatic male adolescents with type 1 diabetes. Congenit Heart Dis (2006) 1:98–103. 10.1111/j.1747-0803.2006.00015.x 18377552

[7] Gupta AK Ravussin E Johannsen DL Stull AJ Cefalu WT Johnson WD. Endothelial dysfunction: an early cardiovascular risk marker in asymptomatic obese individuals with prediabetes. Br J Med Med Res (2012) 2:413–23. 10.9734/BJMMR/2012/1479 22905340 PMC3419538

[8] Konttinen J Lindholm H Sinisalo J Kuosma E Halonen J Hopsu L Association between lowered endothelial function measured by peripheral arterial tonometry and cardio-metabolic risk factors – a cross-sectional study of Finnish municipal workers at risk of diabetes and cardiovascular disease. BMC Cardiovasc Disord (2013) 13:83. 10.1186/1471-2261-13-83 24118794 PMC3852074

[9] Brannick B Wynn A Dagogo-Jack S. Prediabetes as a toxic environment for the initiation of microvascular and macrovascular complications. Exp Biol Med (2016) 241:1323–31. 10.1177/1535370216654227 27302176 PMC4950274

[10] DeFronzo RA Abdul-Ghani M. Assessment and treatment of cardiovascular risk in prediabetes: impaired glucose tolerance and impaired fasting glucose. Am J Cardiol (2011) 108:3B–24B. 10.1016/j.amjcard.2011.03.013 21802577

[11] Li G Zhang P Wang J Gregg EW Yang W Gong Q The long-term effect of lifestyle interventions to prevent diabetes in the China Da Qing Diabetes Prevention Study: a 20-year follow-up study. Lancet (2008) 371:1783–9. 10.1016/S0140-6736(08)60766-7 18502303

[12] Lindstrom J Louheranta A Mannelin M Rastas M Salminen V Eriksson J The Finnish Diabetes Prevention Study (DPS): lifestyle intervention and 3-year results on diet and physical activity. Diabetes Care (2003) 26:3230–6. 10.2337/diacare.26.12.3230 14633807

[13] Ramachandran A Snehalatha C Mary S Mukesh B Bhaskar AD Vijay V. The Indian Diabetes Prevention Programme shows that lifestyle modification and metformin prevent type 2 diabetes in Asian Indian subjects with impaired glucose tolerance (IDPP-1). Diabetologia (2006) 49:289–97. 10.1007/s00125-005-0097-z 16391903

[14] Perreault L Pan Q Mather KJ Watson KE Hamman RF Kahn SE. Effect of regression from prediabetes to normal glucose regulation on long-term reduction in diabetes risk: results from the Diabetes Prevention Program Outcomes Study. Lancet (2012) 379:2243–51. 10.1016/S0140-6736(12)60525-X 22683134 PMC3555407

[15] Dagogo-Jack S Edeoga C Ebenibo S Nyenwe E Wan J Pathobiology of Prediabetes in a Biracial Cohort Research G. Lack of racial disparity in incident prediabetes and glycemic progression among black and white offspring of parents with type 2 diabetes: the pathobiology of prediabetes in a biracial cohort (POP-ABC) study. J Clin Endocrinol Metab (2014) 99:E1078–87. 10.1210/jc.2014-1077 24628558 PMC5393483

[16] Dagogo-Jack S Edeoga C Nyenwe E Chapp-Jumbo E Wan J. Pathobiology of Prediabetes in a Biracial Cohort (POP-ABC): design and methods. Ethn Dis (2011) 21:33–9. 21462727 PMC4841786

[17] Genuth S Alberti KG Bennett P Buse J Defronzo R Kahn R The expert committee on the diagnosis and classification of diabetes mellitus: 2003 follow-up report on the diagnosis of diabetes mellitus. Diabetes Care (2003) 26:3160–7. 10.2337/diacare.26.11.3160 14578255

[18] Dagogo-Jack S Edeoga C Ebenibo S Chapp-Jumbo E. Pathobiology of Prediabetes in a Biracial Cohort (POP-ABC) study: baseline characteristics of enrolled subjects. J Clin Endocrinol Metab (2013) 98:120–8. 10.1210/jc.2012-2902 23118422 PMC3537095

[19] Bonetti PO Pumper GM Higano ST Holmes DR Jr Kuvin JT Lerman A. Noninvasive identification of patients with early coronary atherosclerosis by assessment of digital reactive hyperemia. J Am Coll Cardiol (2004) 44:2137–41. 10.1016/j.jacc.2004.08.062 15582310

[20] Axtell AL Gomari FA Cooke JP. Assessing endothelial vasodilator function with the Endo-PAT 2000. J Vis Exp (2010) 44:2167. 10.3791/2167 20972417 PMC3143035

[21] Nohria A Gerhard-Herman M Creager MA Hurley S Mitra D Ganz P. Role of nitric oxide in the regulation of digital pulse volume amplitude in humans. J Appl Physiol (1985) (2006) 101:545–8. 10.1152/japplphysiol.01285.2005 16614356

[22] Dass N Kilakkathi S Obi B Moosreiner A Krishnaswami S Widlansky ME Effect of gender and adiposity on in vivo vascular function in young African Americans. J Am Soc Hypertens (2017) 11:246–57. 10.1016/j.jash.2017.03.002 28411075

[23] Gargiulo P Marciano C Savarese G D’Amore C Paolillo S Esposito G Endothelial dysfunction in type 2 diabetic patients with normal coronary arteries: a digital reactive hyperemia study. Int J Cardiol (2013) 165:67–71. 10.1016/j.ijcard.2011.07.076 21851998

[24] Lerman A Zeiher AM Endothelial function: cardiac events. Circulation (2005) 111:363–88. 10.1161/01.CIR.0000153339.27064.14 15668353

[25] Maric-Bilkan C. Sex differences in micro- and macro-vascular complications of diabetes mellitus. Clin Sci (Lond) (2017) 131:833–46. 10.1042/CS20160998 28424377

[26] Haynes MP Li L Sinha D Russell KS Hisamoto K Baron R Src kinase mediates phosphatidylinositol 3-kinase/Akt-dependent rapid endothelial nitric-oxide synthase activation by estrogen. J Biol Chem (2003) 278:2118–23. 10.1074/jbc.M210828200 12431978

[27] Guzic-Salobir B Keber I Seljeflot I Arnesen H Vrabic L. Combined hormone replacement therapy improves endothelial function in healthy postmenopausal women. J Intern Med (2001) 250:508–15. 10.1046/j.1365-2796.2001.00910.x 11902819

[28] Shen J Poole JC Topel ML Bidulescu A Morris AA Patel RS Subclinical vascular dysfunction associated with metabolic syndrome in African Americans and whites. J Clin Endocrinol Metab (2015) 100:4231–9. 10.1210/JC.2014-4344 26151335 PMC4702465

[29] Osei K Gaillard T Cook C Kaplow J Bullock M Schuster D. Discrepancies in the regulation of plasma adiponectin and TNF-alpha levels and adipose tissue gene expression in obese African Americans with glucose intolerance: a pilot study using rosiglitazone. Ethn Dis (2005) 15:641–8. 16259488

[30] Boucher AA Edeoga C Ebenibo S Wan J Dagogo-Jack S. Leukocyte count and cardiometabolic risk among healthy subjects with parental type 2 diabetes: the pathobiology of prediabetes in a Biracial Cohort Study. Ethn Dis (2012) 22:445–50. 23140075 PMC4836614

[31] Osei K Gaillard T Schuster D Plasma adiponectin levels in high risk African-Americans with normal glucose tolerance, impaired glucose tolerance, and type 2 diabetes. Obes Res (2005) 13:179–85. 10.1038/oby.2005.23 15761178

[32] Jiang Y Owei I Wan J Ebenibo S Dagogo-Jack S. Adiponectin levels predict prediabetes risk: the pathobiology of prediabetes in A Biracial Cohort (POP-ABC) Study. BMJ Open Diabetes Res Care (2016) 4(1):e000194. 10.1136/bmjdrc-2016-000194 27026810 PMC4800069

[33] Kelly AS Marlatt KL Steinberger J Dengel DR. Younger age is associated with lower reactive hyperemic index but not lower flow-mediated dilation among children and adolescents. Atherosclerosis (2014) 234:410–4. 10.1016/j.atherosclerosis.2014.03.031 24763405 PMC4063084

[34] Jakovljevic DG. Physical activity and cardiovascular aging: Physiological and molecular insights. Exp Gerontol (2017). 10.1016/j.exger.2017.05.016 28546086

[35] Stentz FB Kitabchi AE. Hyperglycemia-induced activation of human T-lymphocytes with de novo emergence of insulin receptors and generation of reactive oxygen species. Biochem Biophys Res Commun (2005) 335:491–5. 10.1016/j.bbrc.2005.07.109 16084832

[36] Boyce G Button E Soo S Wellington C The pleiotropic vasoprotective functions of high density lipoproteins (HDL). J Biomed Res (2017). 10.7555/JBR.31.20160103 PMC6265396 28550271

[37] Aviram M Rosenblat M Bisgaier CL Newton RS Primo-Parmo SL La Du BN Paraoxonase inhibits high-density lipoprotein oxidation and preserves its functions. A possible peroxidative role for paraoxonase. J Clin Invest (1998) 101:1581–90. 10.1172/JCI1649 9541487 PMC508738

[38] Gaillard T Parthasarathy S Osei K HDL dysfunctionality (Paraoxonase) is worse in nondiabetic, postmenopausal African American than in white women. Diabetes Care (2011) 34:e19 10.2337/dc10-1189 21270177 PMC3024386

[39] Healy SJ Osei K Gaillard T. Comparative study of glucose homeostasis, lipids and lipoproteins, HDL functionality, and cardiometabolic parameters in modestly severely obese African Americans and White Americans with prediabetes: implications for the metabolic paradoxes. Diabetes Care (2015) 38:228–35. 10.2337/dc14-1803 25524949 PMC4302264

[40] Heiss C Rodriguez-Mateos A Kelm M. Central role of eNOS in the maintenance of endothelial homeostasis. Antioxid Redox Signal (2015) 22:1230–42. 10.1089/ars.2014.6158 25330054 PMC4410282

[41] Wilson C Lee MD McCarron JG. Acetylcholine released by endothelial cells facilitates flow-mediated dilatation. J Physiol (2016) 594:7267–307. 10.1113/JP272927 27730645 PMC5157078

[42] Beyer AM Freed JK Durand MJ Riedel M Ait-Aissa K Green P Critical role for telomerase in the mechanism of flow-mediated dilation in the human microcirculation. Circ Res (2016) 118:856–66. 10.1161/CIRCRESAHA.115.307918 26699654 PMC4772813

[43] Vincent MA Clerk LH Lindner JR Klibanov AL Clark MG Rattigan S Microvascular recruitment is an early insulin effect that regulates skeletal muscle glucose uptake in vivo. Diabetes (2004) 53:1418–23. 10.2337/diabetes.53.6.1418 15161743

[44] Steinberg HO Chaker H Leaming R Johnson A Brechtel G Baron AD. Obesity/insulin resistance is associated with endothelial dysfunction. Implications for the syndrome of insulin resistance. J Clin Invest (1996) 97:2601–10. 10.1172/JCI118709 8647954 PMC507347

[45] Hogikyan RV Galecki AT Pitt B Halter JB Greene DA Supiano MA. Specific impairment of endothelium-dependent vasodilation in subjects with type 2 diabetes independent of obesity. J Clin Endocrinol Metab (1998) 83:1946–52. 10.1210/jc.83.6.1946 9626124

[46] Balletshofer BM Rittig K Enderle MD Volk A Maerker E Jacob S Endothelial dysfunction is detectable in young normotensive first-degree relatives of subjects with type 2 diabetes in association with insulin resistance. Circulation (2000) 101:1780–4. 10.1161/01.CIR.101.15.1780 10769277

[47] Lteif AA Han K Mather KJ. Obesity, insulin resistance, and the metabolic syndrome: determinants of endothelial dysfunction in whites and blacks. Circulation (2005) 112:32–8. 10.1161/CIRCULATIONAHA.104.520130 15983246

[48] Wendelhag I Fagerberg B Hulthe J Bokemark L Wikstrand J. Endothelium-dependent flow-mediated vasodilatation, insulin resistance and the metabolic syndrome in 60-year-old men. J Intern Med (2002) 252:305–13. 10.1046/j.1365-2796.2002.01036.x 12366603

[49] Muniyappa R Sowers JR. Role of insulin resistance in endothelial dysfunction. Rev Endocr Metab Disord (2013) 14:5–12. 10.1007/s11154-012-9229-1 23306778 PMC3594115

[50] Title LM Lonn E Charbonneau F Fung M Mather KJ Verma S Relationship between brachial artery flow-mediated dilatation, hyperemic shear stress, and the metabolic syndrome. Vasc Med (2008) 13:263–70. 10.1177/1358863X08095154 18940902

[51] Lind L. Endothelium-dependent vasodilation, insulin resistance and the metabolic syndrome in an elderly cohort: the Prospective Investigation of the Vasculature in Uppsala Seniors (PIVUS) study. Atherosclerosis (2008) 196:795–802. 10.1016/j.atherosclerosis.2007.01.014 17335830

